# The Potential of Cannabis in Managing Inflammatory Bowel Disease and Its Future Perspective

**DOI:** 10.7759/cureus.71068

**Published:** 2024-10-08

**Authors:** Arsalan Rauf, Mudassar Nisar, Muhammad Shaeel, Ali Athar, Muhammad Mujtaba Ur Rehman, Filzah Faheem

**Affiliations:** 1 Internal Medicine, Allama Iqbal Medical College, Lahore, PAK; 2 Oncology, George Eliot Hospital, Nuneaton, GBR; 3 Internal Medicine, Shalamar Medical and Dental College, Lahore, PAK; 4 Internal Medicine, Social Security Teaching Hospital, Lahore, PAK; 5 Neurology, AINeuroCare Academy, Dallas, USA

**Keywords:** cannabis, cbd, crohn’s disease, ibd, inflammatory bowel disease, marijuana, ulcerative colitis

## Abstract

Inflammatory bowel disease (IBD) is an inflammation of the gastrointestinal tract mainly categorized as Crohn's disease and ulcerative colitis. The current management of IBD includes pharmacological, surgical, psychological, and complementary treatments, but cannabis effects are also becoming more popular as complementary therapies. Cannabinoids act on two G-protein coupled receptors, CB1 and CB2 located in the brain, enteric nervous system, gastrointestinal cells (epithelial cells), and immune cells. Interaction with these two receptors results in the potential symptomatic and therapeutic effects of cannabis such as decreased gut motility, secretions, and reduced inflammatory edema. Many observational and placebo control trials have been done in the past decades to validate the potential benefits of cannabis in IBD. However, the small sample size of these studies makes it difficult to draw firm conclusions regarding its efficacy and safety. There is a need for large randomized placebo-controlled trials using standardized compositions of cannabinoids with long-term follow-up as cannabis is now an emerging drug to be used for IBD. Future research should emphasize cannabis derivatives and endocannabinoids in order to maximize analgesia and minimize psychotropic side effects.

## Introduction and background

Inflammatory bowel disease (IBD) affects about 0.5% of the population in the Western world with an incidence of 10-30 per 100,000 population. Traditionally, IBD has been associated with white ethnicity, but research has shown that environmental factors are more likely to be a stronger driving force than ethnicity [1]. IBD is defined as an inflammation of the gastrointestinal tract occurring in adolescents and adults. It is further categorized into Crohn’s disease (CD) and ulcerative colitis (UC); both are a form of chronic IBD. UC and CD are more prevalent in North America, England, and Scandinavia than in South Europe, Africa, and Asia [2]. CD can affect any part of the gastrointestinal tract, but it most commonly affects the ileum or perianal region. However, UC mainly starts as inflammation of the rectum spreading proximally in a continuous manner [3].

Current management of IBD includes a combination of pharmacological, surgical, psychological, and complementary therapies. The main goal is to achieve remission, reduce inflammation, and prevent recurrence of the disease. There are several emerging therapies being researched for IBD, including apheresis therapy, intestinal microbiology, stem cell transplants, and exosome therapy, but their application is limited due to a lack of proper research data [4]. There is growing interest in complementary therapies for IBD, including dietary modifications, exercise, probiotics, acupuncture, and meditation, as well as cannabis and turmeric [5]. In this paper, we will discuss the potential of cannabis to reduce gut inflammation and improve pain and related symptoms in IBD patients along with its impact on quality of life.

Cannabis and endocannabinoid system (ECS)

While cannabis became acceptable in Western medicine in the late 19th and early 20th centuries, its active ingredient was not isolated. The first cannabidiol (CBD) isolate from marijuana was identified in 1940, while the first tetrahydrocannabinol (THC) isolate was identified in 1964 [6]. There has been increased interest in studying marijuana's therapeutic effects on IBD over the past few years. Among the 500 potential compounds in cannabis, CBD and THC have been the most extensively studied [7].

**Figure 1:**
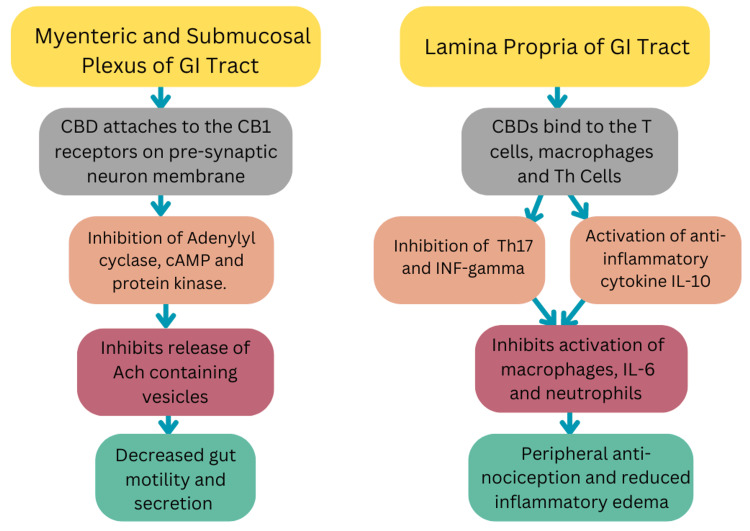
Flowchart of Cannabis Interaction With CB1 and CB2 Receptors. GI: Gastrointestinal tract; CBD: Cannabidiol; cAMP: Cyclic adenosine monophosphate; Ach: Acetylcholine; INF-gamma: Interferon gamma; IL-10: Interleukin 10; IL-6: Interleukin 6 The figure was created by the authors.

The ECS was first discovered in 1968 by scientists Allyn Howlett and W.A. Devane [8]. Cannabinoids act on two G-protein coupled receptors (GPCRs), CB1 and CB2. CB1 receptors are found in the brain (causing psychotropic effects) and the enteric nervous system while CB2 receptors are found in the enteric nervous system, gastrointestinal cells (epithelial cells), and immune cells (macrophages and plasma cells). CB2 receptors are absent in the brain. Interaction with these two receptors results in the potential symptomatic and therapeutic effects of cannabis as shown in Figure 1 [7,9].

Endocannabinoids consist mainly of N-arachidonoylethanolamine (AEA) and 2-arachidonoylglycerol (2-AG), which are lipid signaling molecules. They are synthesized on demand in response to stimuli such as an increase in intracellular calcium levels and stimulation of GPCRs [10]. AEA acts as a partial agonist on both CB1 and CB2 receptors while 2-AG acts as a full agonist with a greater effect on CB1 than CB2. Oleoylethanolamine 9 (OEA), palmitoylethanolamine (PEA), 2-oleoylglycerol (2OG), and 2-palmitoylglycerol (2PG) are also lipid endocannabinoids that may act on receptors other than CB1 or CB2 [11].

Cannabinoid receptors, endogenous ligands, and enzymes responsible for synthesis and degradation of these ligands make up the ECS through which CBD and THC act to implement their therapeutic and psychotic effects.

## Review

Surveys estimate that 11.6 to 15.9% of IBD patients are active cannabis users, while a greater proportion (44.2% to 67%) report ever having used cannabis in their lifetime [12-15]. IBD, known for its debilitating effects, often drives patients to seek symptom relief through complementary medicines like cannabis [14]. Studies show that patients who consume cannabis claim it helps to control the following most commonly cited symptoms: abdominal pain, nausea, anorexia, and diarrhea. 

Therapeutic role of cannabis

Research using murine models of colitis has demonstrated that increased cannabinoid receptor activity correlates with decreased gut inflammation [16-20]. Based on this, a series of observational and trial-based investigations have been done over the last decade or so, evaluating the potential of using cannabis as a therapeutic agent for IBD (Table 1).

**Table 1:** Clinical Studies Showing the Therapeutic Role of Cannabis in Observational and Placebo-Controlled Trials. CD: Crohn’s disease; UC: Ulcerative colitis; n: Number; n/a: Not available; CBD: Cannabidiol; THC: Tetrahydrocannabinol; mg/day: Milligram per day; CDAI: Crohn's disease activity index; PP: Per protocol; HBI: Harvey Bradshaw index; IQR: Interquartile range; CI: Confidence interval; ITT: Intention to treat The table was created by the authors.

Author	Study type	Population	Sample size	Dosing protocol	Findings
Observational Studies					
Naftali et al., 2011 [21]	Retrospective cohort	CD	n = 30	0.5–1.5 mg/day THC	Decrease in the HBI from 14 ± 6.7 to 7 ± 4.7 in 21/30 patients
Naftali et al., 2019 [22]	Prospective cohort	107 CD 20 UC	n = 127	21 mg/day THC 170 mg/day CBD	Decrease in the HBI from 14 ± 6.7 to 7 ± 4.7
Lahat et al., 2011 [23]	Open-label, prospective, single-arm	11 CD 2 UC	n = 13	n/a	Improved quality of life and weight gain; improved disease activity index in CD patients
Dalavaye et al., 2022 [24]	Case series	51 CD 25 UC	n = 76	120 mg/day THC 20.5 mg/day CBD	Improved scores of life quality, anxiety, depression, and sleep
Placebo-Controlled Trials					
Naftali et al., 2013 [25]	Randomized Placebo-controlled	CD	n = 21	230 mg/day THC	Clinical remission THC: CDAI<150 in 5 out of 11 patients Placebo: CDAI<150 in 1 out of 10 patients Clinical response: THC: decrease in CDAI>100 in 10 out of 11 patients Placebo: decrease in CDAI>100 in 4 out of 10 patients
Naftali et al., 2017 [26]	Randomized Placebo-controlled	CD	n = 19	0.3 mg/kg CBD	CBD: CDAI decreased from 337 to 220 Placebo: CDAI decreased from 308 to 216
Naftali et al., 2021 [27]	Randomized Placebo-controlled	UC	n = 32	160 mg/day THC	THC: Disease activity (Lichtiger) scores decreased from 10.9 (IQR 9–14) to 5 (IQR 1–7) Placebo: Disease activity (Lichtiger) scores decreased from 11 (IQR 9–13) to 8 (IQR 7–10)
Naftali et al., 2021 [28]	Randomized Placebo-controlled	CD	n = 56	8 mg CBD and 2 mg THC/day to 320 mg CBD and 80 mg THC/day	CBD/THC: CDAI after eight weeks was 166 (IQR 82–226) Placebo: CDAI after 8 weeks was 237 (IQR 121–271)
Irving et al., 2018 [29]	Randomized Placebo-controlled	UC	n = 62	500 mg/day CBD	ITT analysis: odds ratio = 0.82 (90% CI: 0.29–2.31) PP analysis: Odds ratio = 1.30 (90% CI: 0.42–4.04)

A literature search was conducted on PubMed, Google Scholar, and Cochrane using keywords such as Cannabis, CBD, Cannabidiol, Tetrahydrocannabinol, Marijuana, Inflammatory Bowel Disease, IBD, Crohn's Disease, and Ulcerative Colitis. PubMed showed 276 results related to our research since the year 2010. After a thorough review and removal of duplication, nine studies were selected to be added to the review in which the effects of cannabis were studied in IBD patients. Of these nine studies, four were observational studies while five were randomized controlled trials.

Observational evidence

In 2011, a small yet novel study was conducted by Naftali et al. which involved 30 CD patients who were taking prescription cannabis prior to inclusion and were assessed retrospectively about changes in disease status experienced after starting cannabis [21]. This change was estimated using the Harvey Bradshaw index (HBI), a tool used to stratify the severity of CD based on clinical factors like general well-being and abdominal pain, among others. Most of the study participants inhaled cannabis smoke while one consumed it orally. The study found a significant decrease in the HBI from 14 ± 6.7 to 7 ± 4.7 (P < 0.001) in 21 out of 30 patients. The mean number of bowel movements per day decreased from eight to five. There was also a decreased need for other medications, including steroids.

In 2019, a study by the same author prospectively evaluated the effects of inhaled and oral cannabis in a cohort of 127 IBD patients (107 CD and 20 UC) over a median period of 44 months [22] and found that cannabis therapy was associated with an improved HBI, including the number of bowel movements per day, and reduced need for steroids. Moreover, the cohort patients had an increase in employment rates, and a large proportion of family members expressed satisfaction and perceived benefit from cannabis treatment. Although these external measures of functional improvement were used by the authors primarily to assess addiction-related outcomes, they serve as evidence for demonstrable clinical improvement. Nonetheless, this study did not find any change in inflammatory markers (CRP), which leads one to consider if the observed benefits were due to the tranquilizing effects of cannabis, i.e., a central effect rather than a disease-modifying capability. 

A 2011 prospective study by Lahat et al. evaluated the effects of inhaled cannabis on disease activity and quality of life in 13 patients with IBD [23]. The study reported improvements in general health, daily activities, social functioning, bodily pain, depression, and increased body weight. Daily bowel movements decreased from an average of 5.5 to 3.1. There was also a positive impact on disease activity in CD patients as measured by the partial Mayo score index. A similar investigation done in 2022 by Dalavaye et al. found enhanced quality of life, improved anxiety, depression, and sleep quality after one and three months of treatment with either inhaled or oral cannabis in 76 patients with CD and UC [24].

Placebo-controlled trials

To our knowledge, the first randomized control trial evaluating the effects of cannabis on IBD was conducted by Naftali et al. in 2013 [25]. In this study, 21 patients with CD were randomized to receive inhaled THC-rich cannabis or placebo for eight weeks. The Crohn's disease activity index (CDAI) was used to assess clinical remission, defined as a CDAI score of 150 or less, and clinical response, defined as a 100-point decrease in the CDAI or an improvement in CRP levels or quality of life. Five out of 11 (45%) patients in the cannabis group achieved remission compared to one out of 10 (10%) patients in the placebo group. Response rates were even higher, with 10 patients (90%) in the cannabis group showing a response compared to four (40%) in the placebo group. However, patients did not show an improvement in blood CRP levels. The same group later published a similar trial involving 19 CD patients who were given oral CBD-rich oil or placebo and were followed up for eight weeks [26]. No significant difference in disease activity (mean change in the CDAI) was observed between the two groups. The failure of CBD treatment in this case, as discussed by the authors, could have been due to the following possible causes: the exclusive use of CBD instead of a combination containing other cannabinoids, including THC; low dosing; and using the oral route of administration as opposed to the more common inhaled route. 

In 2021, Naftali and colleagues published two double-blinded placebo-controlled trials. The first trial randomized 32 patients with UC to receive either THC-rich cannabis cigarettes or placebo cigarettes [27]. The second trial involved 56 CD patients treated with either an oral CBD-rich extract or placebo [28]. In both studies, patients on stable chronic medications such as steroids, 5-aminosalicylates, immunomodulators, and biologic agents were permitted to continue these treatments and were asked not to change their medications during the study period. Disease parameters such as clinical symptoms, blood inflammatory markers, and endoscopy findings were evaluated before and after an eight-week treatment period. They found that in both scenarios, cannabis consumption was associated with demonstrable symptomatic benefits, as suggested by reports of improved disease activity and quality of life. However, in neither case did inflammatory markers or endoscopic findings show significant improvement.

Irving et al. conducted a trial with 62 UC patients who received an oral CBD-rich botanical extract or placebo for 10 weeks [29]. Over the course of the trial, a significant number of patients in the CBD group dropped out due to treatment-related adverse effects. This led the authors to perform a per protocol (PP) analysis, which showed that patients who complied with treatment protocols may have had better outcomes in the form of improved disease activity (Mayo scores) and enhanced quality of life. Nonetheless, no difference in fecal calprotectin levels was found despite the PP analysis.

In 2018, Naftali et al. published an abstract of a randomized trial that found evidence of endoscopic improvement with cannabis treatment in patients with UC who were unresponsive to conventional treatment [30]. In addition to being isolated in its claim, data from this trial has not yet been made available for review and thus limits its use as substantiating evidence.

In light of the available data, cannabis use in patients with IBD is likely associated with symptomatic improvement (primarily abdominal pain and number of bowel movements per day), leading to better scores in disease activity and quality of life indices. It is difficult to establish that cannabis administration correlates with any significant reduction in gut inflammation or underlying disease activity. This is also supported by results from two recent systematic reviews and meta-analyses [31,32]. According to two Cochrane reviews, the effects of cannabis on UC and CD are uncertain, and no firm conclusions can be drawn regarding its efficacy and safety [33,34].

Adverse effects and safety 

Current studies on IBD, which use oral and inhaled forms of cannabis, have described a number of adverse events. The 2019 study by Naftali et al. reported the following most common side effects experienced by study participants: dry mouth, memory decline, eye irritation, dizziness, confusion, and restlessness [22]. In the placebo-controlled trials done by Naftali and colleagues, no significant difference in adverse events between treatment and placebo groups was observed [25-28]. Irving et al., who reported a high dropout rate of patients in the CBD group, described 26 (90%) of them as having suffered from adverse effects compared to 15 (48%) of controls [29]. Most of them had nervous or GI system-related side effects such as dizziness, somnolence, nausea, dry mouth, and vomiting, with the majority being mild or moderate in severity.

Recreational cannabis use is associated with a number of short- and long-term consequences for physical and psychological health [35-37], which are thought to be driven by THC, the primary psychotropic component [38]. Short-term effects are underscored by impaired cognition, characterized by a feeling of being “high,” difficulty with memory retrieval, and difficulty learning new tasks [39]. These effects are understood to occur in a dose-dependent manner and are often more intense in infrequent users. While some studies suggest that memory deficits persist in long-term users [40], others report their absence [41]. A meta-analysis examining 11 studies found that long-term cannabis use was associated with very subtle deficits in memory and learning, but the effect sizes were so small that the authors questioned the real-life impact of these findings [42].

Evidence suggests that cannabis and its active component, THC, negatively impact problem solving and abstract reasoning [43,44]. There also seems to be a reasonable association between THC and increased disinhibition and impulsivity [45,46]. A dispute exists over whether or not cannabis is linked to impaired decision-making and risk-taking behaviors. 

Long-term use of cannabis is strongly related to addiction risk, which is estimated to be nine percent [47]. Individuals who use cannabis regularly and initiate it at an earlier age are at higher risk. Consistent use leads to withdrawal symptoms, making cessation challenging. Cannabis also has a reputation as a gateway substance, potentially paving the way for further drug addictions. In the 2019 study by Naftali and colleagues, as previously alluded to, participants in the cannabis group did not show signs of developing addiction. Rather, they displayed improvement in day-to-day functioning, as shown by their employment and social outcomes [22]. 

Cannabis has also been shown to be associated with an increased risk of stroke and possibly myocardial infarction but the effect of cannabis on platelet aggregation was inconclusive [48]. Smoking cannabis leads to respiratory symptoms (cough, sputum production, and dyspnea) and increases the risk of pulmonary infections [49,50]. One of the most common GI-related side effects is hyperemesis syndrome [51,52]. This disorder is characterized by cyclic episodes of nausea and vomiting occurring in chronic cannabis users, who often find that taking hot showers relieves these symptoms. Cannabis has been demonstrated to negatively impact male and female fertility [53,54]. It is also linked to adverse birth outcomes because of its negative effects on fetal neurodevelopment [55,56].

Research has associated cannabis with the development of both acute and chronic psychotic symptoms. Healthy subjects exposed to THC have been shown to experience transient positive and negative symptoms and perceptual alterations, along with general neurocognitive impairment [44,57]. Cannabis consumption greatly elevates the likelihood of psychotic illnesses, with higher doses leading to greater risk [58].

Although recreational cannabis is known to be strongly associated with a wide variety of short- and long-term effects, limited studies on IBD have not reported any serious adverse events. The follow-up duration was limited; hence, it is difficult to draw conclusions regarding the long-term effects of medicinal cannabis.

Regulation of cannabis usage

Cannabis and cannabis resins were classified as schedule one and four substances according to the Drug Control Regimen of 1961 Convention. The facilitation of cannabis for medical and research purposes was highlighted in 2016 by the UN Assembly. Later on, in 2019 WHO’s Expert Committee on Drug Dependence (ECDD) not only recommended removing cannabis from Schedule four drug list but also clarified that cannabidiol-based preparations without THC are not subject to international drug control, as cannabidiol is not a psychoactive substance and does not have a high potential for abuse or dependence. According to the ECDD's recommendation, the UN Commission on Narcotic Drugs (UN CND) decided to follow it in December 2020 [59]. In January 2023, the FDA provided final regulation guidance for use of cannabis and cannabis-related products for clinical research and resources to assist with quality considerations and control status. According to the guidelines, clinical research may use cannabis sources with 0.3 percent delta-9 THC or more on a dry weight basis if the FDA deems them of adequate quality after reviewing an investigational new drug. If it exceeds 0.3 percent Delta-9 THC by dry weight then sponsors are required to conform to CSA (Controlled Substances Act) and DEA (Drug Enforcement Administration) requirements when used as investigational drugs [60].

Out of 193 sovereign states recognized by the United Nations, approximately 36 countries (19 percent) have regulatory frameworks for medical cannabis in place, four countries (two percent) have exception laws that provide access to medical cannabis, and 16 countries (eight percent) are developing or regulating their own laws [59]. In the United States, 37 states, four territories, and the District of Columbia allow medical cannabis use, while three states and one territory prohibit it. Some cheaper CBD products aren't as reliable as others due to mislabeling concerns about the exact content of the product [61]. In Europe, consumption of cannabis is considered a serious offense, punishable by imprisonment in some countries Cyprus, France, Finland, Greece, Hungary, Norway, Sweden, and Türkiye, but possession for medical and scientific reasons is permitted. Likewise, unauthorized possession of cannabis can result in imprisonment in countries like Norway, Sweden, Finland, the United Kingdom, Denmark, Estonia, Poland, Germany, France, Austria, Hungary, Romania, Slovakia, Czech Republic, and Greece [62-64].

Current challenges and future perspectives

Although there is a growing interest in exploring the role that cannabis can play in helping to manage IBD, regulatory obstacles in making cannabis and its products legally available as medicinal agents exist worldwide [59]. Of the studies that were able to investigate its role, follow-up durations were short, and thus very little can be said about the long-term consequences of using cannabis in patients with IBD. Additionally, it seems there is very little conclusive information available in current studies on what is considered appropriate or most effective and safe when it comes to the formulations, dosages, and delivery modalities of cannabis [65]. Hence, there is a need for large randomized placebo-controlled trials using standardized compositions of cannabinoids with long-term follow-up.

Research conducted on therapeutic applications of cannabis in recent years suggests that it may soon become an important symptom-controlling drug. In a recent FDA approval, Epidiolex®, the first drug to contain active cannabis ingredients, was approved for treating seizures. As of yet, Sativex® is not approved for use in the United States, but it has been approved in Canada, New Zealand, and 21 European countries, including the UK to treat multiple sclerosis and pain in cancer patients [64]. To maximize analgesia while minimizing psychotropic side effects, future research should primarily focus on cannabis derivatives and endocannabinoids. Researchers will likely examine the long-term effects and tolerance of cannabis use in patients with IBD.

Limitations

The limited number of studies being included in the review increases the risk of bias thereby diminishing the robustness of our findings. Also the small sample size further undermines the reliability of the results. That’s why large-scale observational and randomized control trials are needed to fully understand the therapeutic implications of cannabis. Additionally, few studies reported adverse effects due to the short follow-up periods, highlighting the need for more research specifically focused on the long-term adverse effects of cannabis.

## Conclusions

Many IBD patients use cannabis to control disease symptoms, and there is emerging evidence that it may play a role in disease management. Current studies have found cannabis to improve quality of life and symptoms, whereas there is no compelling evidence to suggest that it reduces gut inflammation or the underlying disease process. Large randomized controlled trials with long-term follow-up are required to evaluate the anti-inflammatory role of cannabis in IBD.
